# Serum Th1, Th2, Th17, and innate immune system biomarkers are elevated in pediatric alopecia areata with and without concurrent atopic dermatitis: A cross-sectional study

**DOI:** 10.1016/j.jdin.2024.09.008

**Published:** 2024-10-23

**Authors:** Ryan S.Q. Geng, Katherine A. Buhler, May Y. Choi, David Croitoru, Elena Pope, Marvin J. Fritzler, Cathryn Sibbald

**Affiliations:** a Temerty School of Medicine, University of Toronto, Toronto, Ontario, Canada; b Cumming School of Medicine, University of Calgary, Calgary, Alberta, Canada; c Division of Dermatology, Department of Paediatrics, The Hospital for Sick Children, University of Toronto, Toronto, Ontario, Canada

**Keywords:** alopecia areata, atopic dermatitis, biomarkers, pediatric dermatology

*To the Editor:* Alopecia areata (AA) is a nonscarring alopecia with an autoimmune etiology, where aberrant T-lymphocyte mediated immune responses are implicated in its pathogenesis.^1^ Several robust studies have reported elevated T_h_1 (interferon gamma, CXCL9/10/11, interleukin 2 receptor antagonist [IL-2RA], IL-12), T_h_2 (IL-10/13, CCL7/11/13/17/22/23, CCR4), T_h_17 (CCL20, PI3, S100A12), and innate (IL-6/8) immune biomarkers.^2^, ^3^, ^4^ Differences in biomarker profiles have been observed in patients with AA and concomitant atopy. IL-13 was only elevated in atopic AA, whereas interferon gamma was only elevated in nonatopic AA, suggesting heterogeneity among patients with AA.^3^ However, studies have yet to report biomarker profiles in pediatric patients with AA. With increasing prevalence of AA in pediatric patients,^5^ a cross-sectional pilot study was conducted to investigate the biomarker profiles of pediatric patients with AA alone and AA with concomitant atopic dermatitis (AD).

Patients with AA of 6 to 18 years with >30% scalp involvement off systemic anti-inflammatory therapies for 3 months (AA alone, *n* = 10; AA + AD, *n* = 10) and age-matched healthy controls (*n* = 10) were enrolled. AD was diagnosed by a pediatric dermatologist and severity assessed by Eczema Area and Severity Index, with mild-moderate disease (Eczema Area and Severity Index <21) being included. Proteomic analysis of patient plasma was conducted for 240 cytokines using bead-based multiplex technology (MilliporeSigma). Cytokine levels between the 3 groups were compared by 1-way analysis of variance.

All 3 groups consisted of the same number of male and female patients, with no significant differences in Severity of Alopecia Tool score and disease duration of AA between the AA alone and AA + AD groups (Table I).

**Table I:** Demographic and disease characteristics of patients included in the study

Characteristics	AA alone (*n* =10)	AA + AD (*n* = 10)	Controls (*n* = 10)
Age (y)	10.90 ± 3.60	11.10 ± 3.38	12.60 ± 3.34
Sex (% female)	50% (5/10)	50% (5/10)	50% (5/10)
Ethnicity	Asian: 50% White: 30% Black: 20%	Asian: 40% White: 30% Black: 10% Hispanic: 10%	Asian: 40% White: 50% Black: 10%
SALT score	77.00 ± 27.81	65.50 ± 23.15	-
Eyebrow involvement	Complete loss: 20% Partial loss: 30% No involvement: 50%	Complete loss: 20% Partial loss: 40% No involvement: 40%	-
Eyelash involvement	Complete loss: 20% Partial loss: 20% No involvement: 60%	Complete loss: 20% Partial loss: 30% No involvement: 50%	-
AA current episode duration (mo)	28.10 ± 22.46	23.10 ± 27.00	-

Values are reported as mean ± SD, unless otherwise indicated.

*AA*, Alopecia areata; *AD*, atopic dermatitis; *SALT*, Severity of Alopecia Tool.

Elevations in T_h_1 (sIL-2RA, IL-1α/β), T_h_2 (CCL17, IL-1RA, IL-4), T_h_17 (IL-17A/22/23), innate (IL-8, B-cell activating factor, C-reactive protein, granulocyte colony-stimulating factor, matrix metalloproteinase 9) biomarkers, and vascular endothelial growth factor A were observed (Fig 1). Additionally, Severity of Alopecia Tool scores correlated significantly with higher IL-1α (*P* < .01), IL-1β (*P* < .01), IL-1RA (*P* < .01), IL-17A (*P* < .01), and IL-8 (*P* < .05). However, no significant correlation between alopecia duration was observed with any of the elevated cytokines.

**Fig 1:**
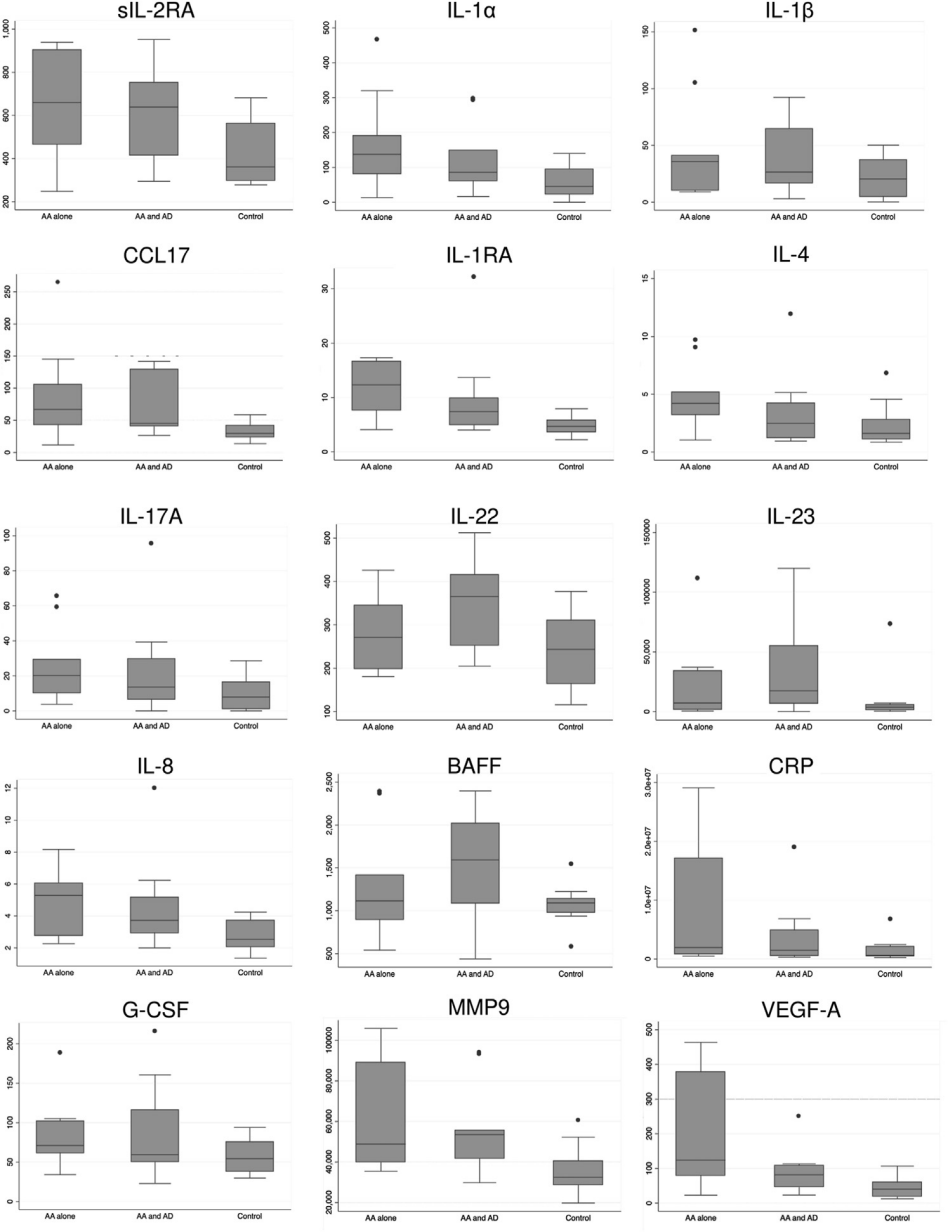
Th1, Th2, Th17, and innate immune system biomarkers identified in this study with significant differences between AA alone, AA + AD, and healthy controls by 1-way analysis of variance (*P* < .1). The dots represent data points outside of the IQR. *AA*, Alopecia areata; *AD*, alopecia/dermatitis; *BAFF*, B-cell activating factor; *CCL*, C-C motif chemokine ligand; *CRP*, C-reactive protein; *G-CSF*, granulocyte colony-stimulating factor; *IL*, interleukin; *MMP*, matrix metalloproteinase; *RA*, receptor antagonist; *VEGF*, vascular endothelial growth factor.

Our study adds insight into biomarker profiles of pediatric patients with AA. Compared with the cross-sectional study of blood biomarkers in adult patients with AA by Glickman et al,^3^ we similarly observed elevations in sIL-2RA, CXCL9, CCL17, IL-8, and matrix metalloproteinase 9. However, we did not observe elevations in CXCL11, CCL7, IL-6, and interferon gamma.^3^ These differences noted in our study could reflect variations in biomarker profiles between adult and pediatric patients with AA, but more research is needed to confirm and explain any difference in pediatric and adult pathogenesis. Heterogeneity was also observed among the pediatric patients in our study. Patients with AA + AD trended toward higher levels of T_h_17 cytokines, especially IL-23, compared with patients with AA alone. Patients with AA alone trended toward higher levels of IL-1α and IL-1RA compared with patients with AA + AD. These observations support the idea that AA is not a homogenous group and there may be different AA subsets with distinct biomarker profiles. It would be interesting to evaluate if patients with elevations in certain biomarkers respond differently to targeted therapy compared with patients without. Further studies assessing longitudinal changes in biomarker profiles in patients on therapy are required to identify biomarkers with prognostic utility.

Limitations of our study include small sample size and insufficient statistical power to conduct pair-wise comparisons.

## Conflicts of interest

Dr Sibbald has received honoraria from AbbVie, Leo, Pfizer, Miravo, Novartis, UCB, and Sanofi/Regeneron, unrelated to this work. Dr Fritzler has received consulting and speaking honoraria from Werfen, unrelated to this work. Dr Pope has received honoraria from AbbVie, Leo, Pfizer, Sanofi, and Timber Pharmaceuticals and research grants from Timber Pharmaceuticals, unrelated to this work. Dr Choi has received consulting and speaking honoraria from AstraZeneca, GSK, Mallinckrodt Pharmaceuticals, MitogenDx, Werfen, Celltrion, and Organon. Dr Croitoru has received honoraria from AbbVie, Amgen, Bausch Health, Boehringer Ingelheim, Novartis, Pfizer, Sanofi-Regeneron, and UCB, unrelated to this work. Authors Geng and Buhler have no conflicts of interest to declare.
